# Seasonal variation in telomerase activity and telomere dynamics in a hibernating rodent, the garden dormouse (*Eliomys quercinus*)

**DOI:** 10.3389/fphys.2023.1298505

**Published:** 2023-11-22

**Authors:** Carlos Galindo-Lalana, Franz Hoelzl, Sandrine Zahn, Caroline Habold, Jessica S. Cornils, Sylvain Giroud, Steve Smith

**Affiliations:** ^1^ Department of Interdisciplinary Life Sciences, Konrad Lorenz Institute of Ethology, University of Veterinary Medicine, Vienna, Austria; ^2^ University of Strasbourg, Centre National de la Recherche Scientifique, Institut Pluridisciplinaire Hubert Curien, Strasbourg, France; ^3^ Research Institute of Wildlife Ecology, Department of Interdisciplinary Life Sciences, University of Veterinary Medicine, Vienna, Austria; ^4^ Energetics Lab, Department of Biology, Northern Michigan University, Marquette, MI, United States

**Keywords:** torpor, euthermia, oxidative damage, biological ageing, somatic maintenance

## Abstract

Telomere dynamics in hibernating species are known to reflect seasonal changes in somatic maintenance. Throughout hibernation, the periodic states of rewarming, known as inter-bout euthermia or arousals, are associated with high metabolic costs including shortening of telomeres. In the active season, if high energetic resources are available, telomere length can be restored in preparation for the upcoming winter. The mechanism for telomere elongation has not been clearly demonstrated, although the action of the ribonucleoprotein complex, telomerase, has been implicated in many species. Here we tested for levels of telomerase activity in the garden dormouse (*Eliomys quercinus*) at different seasonal time points throughout the year and across ages from liver tissues of male juveniles to adults. We found that telomerase is active at high levels across seasons (during torpor and inter-bout euthermia, plus in the active season) but that there was a substantial decrease in activity in the month prior to hibernation. Telomerase levels were consistent across age groups and were independent of feeding regime and time of birth (early or late born). The changes in activity levels that we detected were broadly associated with changes in telomere lengths measured in the same tissues. We hypothesise that i) telomerase is the mechanism used by garden dormice for maintenance of telomeres and that ii) activity is kept at high levels throughout the year until pre-hibernation when resources are diverted to increasing fat reserves for overwintering. We found no evidence for a decrease in telomerase activity with age or a final increase in telomere length which has been detected in other hibernating rodents.

## Introduction

Understanding the complexity of telomere dynamics in non-model organisms has become a burgeoning field of research in ecology and evolution (see reviews in (Monaghan and Ozanne, 2018; Smith et al., 2022). In contrast to early research in humans and cancer models, where telomeres typically shorten with age as a result of the end replication problem and oxidative stress (Harley et al., 1990; Blasco, 2007; Aubert, 2014), recent research has shown that in some organisms telomeres can lengthen as individuals become older and that telomeres can even have a complex and dynamic role in life histories (Monaghan and Ozanne, 2018; Marasco et al., 2022). Such findings of active telomere maintenance struggled to find traction initially but repeated examples across many taxa have led to a general acceptance that the process is not uncommon non-model species (Hoelzl et al., 2016b; Criscuolo et al., 2020; Panasiak et al., 2020; Tissier et al., 2022; Giroud et al., 2023). The underlying mechanisms for telomere repair, however, remain poorly understood.

Telomeres in hibernating rodents, particularly, have been shown to cycle through periods of loss and periods of maintenance (Hoelzl et al., 2016a; Giroud et al., 2023). The gain and loss of telomeres seems tightly bound to seasonal variation in energy availability and to phases characterized by marked changes in metabolic demands, such as those observed during torpor and hibernation (Hoelzl et al., 2016a). Active periods are time points when telomeres can be elongated but the underlying process for elongation is unclear and seemingly energy costly. In a supplemental feeding experiment on edible dormice (*Glis glis*) it was shown that elongation was possible in the active season but only when extra food resources were made available in the form of energy rich sunflower seeds (Hoelzl et al., 2016a). Similarly, telomere elongation is possible in hibernating garden dormice (*Eliomys quercinus*) if food is available (Giroud et al., 2023). Telomerase, the ribo-nucleoprotein complex, is a prime candidate as the mechanism for telomere extension but little is known of its activity in non-model species. In experimental settings telomerase has been shown to act to increase telomere length in these examples (de Jesus et al., 2011; Reichert et al., 2014; Tomás-Loba et al., 2008; see review here; Criscuolo et al., 2018).

Telomerase activity (TA) is problematic to measure in many wild populations due to difficulties in collecting appropriate samples and processing them in a time-efficient manner. The situation is compounded by the lack of knowledge about when and how long telomerase should be active to generate a detectable increase in telomere length. It is vitally important to resolve this problem to allow researchers to disentangle the complex mechanisms of telomere maintenance and the relationships with energy balances that may constrain life histories. Previous research has shown that hibernating rodents suffer a loss of telomere length throughout hibernation and that this loss is intricately linked to the number and length of interbout euthermia phases (IBE) across the hibernation period (Hoelzl et al., 2016a). All hibernation periods are typified by periods of extreme reduction in metabolism and body temperature (torpor) interspersed with short bursts of euthermia where individuals return to normal metabolism and body temperature before returning into the torpor phase (Lyman et al., 1982; Carey et al., 2003; Heldmaier et al., 2004; Hoelzl et al., 2015). Telomere attrition across hibernation has been shown in both edible dormice (*Glis glis*) and garden dormice (*E. quercinus*) as well as in other hibernating rodents such as Columbian ground squirrels (*Urocitellus columbianus*), eastern chipmunks (*Tamias striatus*), and also in some bat species (Hoelzl et al., 2016b; Foley et al., 2018; Nowack et al., 2019; Giroud et al., 2022; Tissier et al., 2022; Viblanc et al., 2022). In garden dormice however, individuals were able to maintain or increase relative telomere length (RTL) during hibernation when provided with food *ad libitum* (Giroud et al., 2023). Further, Hoelzl et al. (2016a) showed that telomere loss was most closely related to the number of IBE episodes during hibernation. Combined, this evidence suggests that IBE is highly energy demanding and that it involves bursts of reactive oxygen species (ROS) generation that are hugely damaging to telomeres. It further suggests that telomerase activity is reduced throughout hibernation and thus telomere repair does not take place during this period at least when animals lack food supply.

The most likely candidate period for telomere maintenance is during the early active season when energy is abundant and individuals emerge from the period of high ROS damage during hibernation. Alternatively, telomere maintenance may occur immediately prior to hibernation when body fat reserves are increasing dramatically in preparation for the winter months. Although, during this time the costs of lipogenesis are likely very high and may not allow investment into telomere maintenance and increased TA, especially in the liver. While research has shown that telomeres can be elongated in edible dormice in the active season when food availability is high (Hoelzl et al., 2016a) and also with chronological age in Eastern chipmunks (Tissier et al., 2022), telomerase activity was not measured in the same individuals, so the mechanism for telomere repair is still unclear. Sampling individuals throughout the entire active season and also throughout hibernation, including during deep torpor and IBE, and measuring telomerase activity as well as telomere length, is the only way to resolve the timing and likely mechanism of telomere maintenance. Such sampling requires a well-managed population where all individuals are monitored for age, body mass, body temperature and food intake.

In this study, we used a long-term captive colony of garden dormice as an experimental system to understand the timing and activation of telomerase and how it relates directly to telomere maintenance. By using a closely monitored population we were able to very accurately determine the state of individuals throughout hibernation as well as their body mass and energy intake throughout the active season. The colony, housed at the Research Institute of Wildlife Ecology in Vienna, contained individuals of different ages and sexes, from which life-history parameters could be closely monitored. By sampling liver tissues of individuals at the distinct timepoints of interest, we were able to measure both the level of TA relative to total protein amount as well as RTL of the very same tissue samples. We made measurements of RTL and TA from liver tissues as this organ has been shown in pilot studies to exhibit high levels of TA and is expected to be highly active during lipogenesis and “lipid-mobilisation” to provide energy during hibernation. This provides a very powerful method to determine the interplay between TA, RTL, season and age while accounting for sex and body mass in the same models. We hypothesise that TA will be low throughout all points during hibernation and will peak early in the active season, when food availability is high, but will decrease substantially pre-hibernation when individuals are building fat reserves. Due to high metabolic demands in the liver (lipogenesis), increased foraging activity and associated extensive ROS production, telomeres are not likely to be elongated in autumn. We therefore expect a cyclical seasonal dynamic for RTL with loss over winter and a regeneration early in the active season.

The advantage of our well monitored study system is that we have a deep knowledge of their physiology as well as detailed data on the use of torpor and IBE, food intake, age, and body mass of all sampled individuals. Further by measuring RTL and TA from the same tissues, we can access simultaneous estimates of these inter-related parameters to clearly assess the effect of enzymatic repair on telomeres.

## Materials and methods

### Ethical note

All procedures have been discussed and approved by the institutional ethics and animal welfare committee in accordance with GSP guidelines and national legislation (ETK-046/03/2020, ETK-108/06/2022, and ETK-150/09/2022), and the national authority according to §§29 of Animal Experiments Act, Tierversuchsgesetz 2012 - TVG 2012 (BMBWF-68.205/0175-V/3b/2018).

### Study species and sampling

The samples for this study came from an ongoing project on torpor use in garden dormice and were made available for assessment of TA and RTL at relevant seasonal timepoints (early active season, pre-hibernation, torpor bout in hibernation season, IBE in hibernation season). Due to logistic constraints, only juvenile male individuals were available and monitored over the hibernation season but both sexes as adults were available for measurements over the active season.

We used 107 individuals in this study including 80 (43 females and 37 males) adult garden dormice from the active season (including the early active season and the pre-hibernation fattening phase), ages ranging between 11 and 62 months and having undergone at least one complete hibernation; and a further 27 juvenile males sampled during their first hibernation (13 in torpor and 12 in IBE) at the age of 10 months. These groups will be called “adult” and “juvenile” respectively for the remainder of the text. Adult individuals in the early active season group contained animals that were born early in the season (March/April) and others that were late born (July). All individuals were fed *ad libitum* except for 13 juvenile individuals in the hibernation group that were on a food restriction regime (see Supplementary Table S10). Of the active season individuals, the animals were born in captivity and raised under natural climatic conditions in outdoor enclosures at the Research Institute of Wildlife Ecology (FIWI) of the University of Veterinary Medicine Vienna, Austria (48°15′N, 16°22′E). The study individuals were euthanized and the liver was flash frozen in liquid nitrogen within 5 min after death of the animals. All samples were stored at −80°C upon processing.

Torpid animals were sacrificed by immediate decapitation. Euthermic animals during hibernation, pre-hibernation and summer were euthanized by incrementally exposure to carbon dioxide (CO2, 100%) until loss of consciousness followed by decapitation, as previously described (Huber et al., 2021). Once individuals were euthanized, the liver was flash frozen in liquid nitrogen within 5 min after death of the animals. All samples were stored at −80°C upon processing.

### Temperature recording and torpor pattern

We used nest temperature as a proxy for T_b_ to estimate torpor use, as described by Willis et al. (2005) and used in previous studies in garden dormice (Giroud et al., 2012; Giroud et al., 2014; Mahlert et al., 2018; Nowack et al., 2019). In brief, the nest box was equipped with a customized temperature data logger (FIWI, Vienna, Austria; resolution: 0.2°C, accuracy: ±0.06°C), which recorded the temperature of the nest every minute. Nests were big enough for one dormouse to fit completely inside but small enough that it had to sit directly on the thermologger. The floors of the nests were covered with a thin layer of hay to provide familiar nesting conditions but to still ensure the contact between the animal and data loggers. To mimic normal conditions during hibernation, animals were kept under constant darkness and at a near stable temperature in the cooling units. In their housing setup dormice were exposed to environmental temperatures of 13.5±3.8°C at pre-hibernation and 24.5±1.8°C during the summer just prior to and during sacrifices.

### Molecular procedures

A small portion of liver tissue was harvested from euthanized individuals as part of standard colony management measures. For the TA and RTL analysis, samples were stored immediately at −80°C until analysis. To estimate TA we used a modified version of the droplet digital telomere repeat amplification protocol (ddTRAP) of (Ludlow et al., 2014). In brief, approximately 0.5 mg of frozen liver was powdered in liquid nitrogen for 20 s at 50 osc/sec in a Tissue Lyser (Qiagen, Germany). TRAP lysis buffer (80 μL) was added to each sample and incubated on ice for 2 hours (10 mM Tris-HCl, pH 8.0, 1mM MgCl_2_, 1 mM EDTA, 1% (vol/vol) NP-40, 0.25 mM sodium deoxycholate, 10% (vol/vol) glycerol, 150 mM NaCl, 5 mM *β*-mercaptoethanol; 0.1 mM AEBSF (4-(2-aminoethyl)benzenesulfonyl fluoride hydrochloride). One microlitre of lysed sample was then used in the TRAP reaction containing 1x TRAP buffer (10x concentration: 200 mM Tris-HCl, pH 8.3, 15 mM MgCl_2_), 0.4 mg/mL BSA, TS primer (HPLC purified, 200nM; 5’ AAT​CCG​TCG​AGC​AGA​GTT), dNTPs (2.5 mM each) and purified water to a volume of 50 μL. The TRAP reaction was performed in a thermal cycler with the lid heating deactivated. To test if temperature had an effect on TA we incubated the lysate at 25°C (as in Ludlow et al., 2014) and 5°C (to mimic torpor body temperature) for 40 min and then heated to 95°C for 6 min before cooling to 4°C. Two μl of the TRAP product was then added to the ddPCR reaction mix containing 1xEvaGreen ddPCR Supermix v2.0 (Bio-Rad, Hercules, CA, United States), 50  nM TS primer, 50 nM of the ACX primer and purified water to a volume of 20 μL. After droplet generation (QX200 drop generator, Bio-Rad), 40 μL of the generated emulsion was transferred to a 96-well PCR plate (twin-tec 96-well plate, Eppendorf, Fisher) and sealed with foil (Thermo Scientific, AB0757). PCR was performed (GeneAmp^®^ PCR System 9,700, Applied Biosystems, Foster City, CA) with a temperature increase of 2.5°C/s between all steps. Activation of Taq polymerase (95°C for 5 min) was followed by 40 cycles of 95°C for 30s, 54°C for 30 s, 72°C for 30 s, then held at 12°C. Fluorescence was read on the droplet reader (QX200, Bio-Rad) after PCR (using the 6-Fam channel Nucleic Acids Research, 2014 e104 (channel 1 on the software)). One no-template lysis buffer control sample and one control sample without primers but with lysate were added to determine the threshold between positive and negative results. The output was given in number of molecules of extension products per microliter of ddPCR reaction, indicating the number of extended TS molecules and therefore telomerase activity. All values were normalised to total protein content in each sample (a proxy for the number of cells in each assay) as determined via a standard Bradford assay of 30 μL of the remaining lysate.

DNA was extracted from the same lysate generated for the ddTRAP measurement of TA. To 30 μL lysate we added 500 µL TNES buffer and 15 µL Proteinase K. Samples were vortexed and incubated for 20 min at 55°C while shaking. Then 180 µL of 5M NaCl was added before vortexing and centrifugation for 20 min at 12,000 rpm. Supernatant was transferred to a new 1.5 mL tube (containing 1 µL Glycogen). Precipitation was performed with chilled isopropanol incubated at −80°C for 30 min. DNA was then pelleted and resuspended in 100 μL TE buffer. Concentration and quality (260/280 ratio) of the extracted DNA was determined using a NanoDrop 2000c (Peqlab Biotechnologie GmbH, Erlangen, Germany).

#### Relative telomere length

An 54 bp portion of the cMYC proto-oncogene was used as the non-variable copy number (non-VCN) gene. Primer sequences for the non-VCN gene were 5‘-GAG GGC CAA GTT GGA CAG TG-3‘(cMYC_F) and 5’-TTG CGG TTG TTG CTG ATC TG-3’ (cMYCR), and telomeric primer sequences were 5′-CGG TTT GTT TGG GTT TGG GTT TGG GTT TGG GTT TGG GTT-3′ (tel 1b) and 5′-GGC TTG CCT TAC CCT TAC CCT TAC CCT TAC CCT TAC CCT-3′ (tel 2b) RTL was estimated via qPCR as described by Hoelzl et al. (2016b). Non-VCN gene and telomere PCRs were carried out in separate runs with 20 ng DNA per reaction, 400 nmol l−1 of each primer in a final volume of 20 μL containing 10 μL of Promega BRYT Green GoTaq^®^ qPCR Master Mix (Cat. Nr. A6001/2; Promega, Madison, United States). PCR conditions for cMYC were 10 min at 95°C followed by 40 cycles of 10 s at 95°C, 20 s at 63°C and 20 s at 72°C. PCR conditions for the telomere primers were 10min at 95°C followed by 40 cycles of 10 s at 95°C, 20 s at 56°C and 20 s at 72°C. In each run, a final melting step was included with the temperature ramping from 65°C to 95°C, at 1°C steps. Two reference standard samples (standard A and standard B) were included in every run and compared with all ratios of telomere to non-VCN gene. A non-template control was included as well in every run. To minimize pipetting errors, reactions were prepared using the Qiagility PCR robot (Qiagen, Germany). Cycling was conducted on a Rotorgene Q quantitative thermocycler (Qiagen, Germany). For analysis of the non-baseline corrected raw qPCR data, the software LinRegPCR (2012.0) was used. RTL was calculated using a modified formula from Ruijter et al. (2009), where E is the qPCR efficiency and Ct the cycle threshold. The subscript ST refers to the telomere reaction of the standard sample, SC to the control gene reaction of the standard sample, T to the telomere reaction of the target sample and C to the control gene (cMYC) reaction of the target sample: RTL = (E_T_^CtT^/E_ST_^CtST^)/(E_c_^CtC^/E_SC_^CtSC^).

The mean qPCR efficiency was calculated via the amplification plot method (Ramakers et al., 2003) which gives lower but more accurate estimates of efficiency than standard curve based methods (Morinha et al., 2020; Spießberger et al., 2022). For the non-VCN gene and telomere reactions, mean qPCR efficiencies were 91.0% and 78.6%, respectively.

### Statistics

All analyses were carried out in R 4.3.1 (R Core Team, 2023). A Wilcoxon rank test was performed on the assay temperature comparison between TA at 5°C and 25°C. For comparison between groups the package emmeans was used (Lenth, 2023). Analyses were split in two groups adult females and males in the active and prehibernation season and juvenile male garden dormice during hibernation in torpor and IBE. In case of the adult/yearling group, we used Generalized linear models to analyze the influence of the state (active/prehibernation), body mass, sex, age in month and the time point of birth (early/late) in the season as explanatory variables for the square root transformed TRAP assay concentration at 25°C (Gamma distribution), as well as the RTL ratio (Gaussian distribution) in a separate model. For the juvenile garden dormice, we used the state (torpor/IBE), body mass and the food regime (*ad libitum*/restricted) to explain TRAP assay concentration at 25°C (Gaussian distribution) and RTL ratio (Gamma distribution). We checked regression diagnostics with the built-in visual inspection function in base R 4.3.1 (R Core Team, 2023). The model with the best fit, was chosen by comparing AICc values for different model specifications, the models with the lowest AICc, per analysis, were used for calculation of the Relative Variable Importance (Barton, 2023). Variables with values over 0.7 for RVI were defined as being important for the respective model. In the case of the TRAP assay model for the adult animals, we had one case with no change in TRAP assay concentration, to avoid losing samples for modeling we added 0.001 to all TRAP assay concentration values, because for the Gamma distribution exclusively positive values are needed. We used the ggeffects package (Lüdecke, 2018) to create the estimated marginal means at the specific values of the model terms, to create partial plots (Figure 3).

## Results

Out of 107 animals in the study (Table 1), for the ddTRAP assay, six adults failed (three females in prehibernation, and one female in the active season; one male in prehibernation, and one in the active season), and two juveniles failed (one male in torpor, and one male in IBE). A further two adults failed for the RTL analysis (one female and one male, both in prehibernation). This left data for 99 individuals for the ddTRAP assay (74 adults and 25 juveniles) and for 105 individuals for RTL (78 adults and 27 juveniles).

**TABLE 1:** Summary of individuals analysed for telomerase activity (TA) and relative telomere length (RTL) from the study colony of garden dormice at the Research Institute for Wildlife Ecology, Vienna.

	Early active season				Prehibernation		Torpor	IBE
	Male		Female		Male	Female	Male	
	Early born	Late born	Early born	Late born	Early born			
Adults (*ad libitum*)	8	6	12	8	23	23	-	-
Juveniles (*ad libitum*)	-	-	-	-	-	-	8	6
Juveniles (fasted)	-	-	-	-	-	-	7	6

**FIGURE 1:**
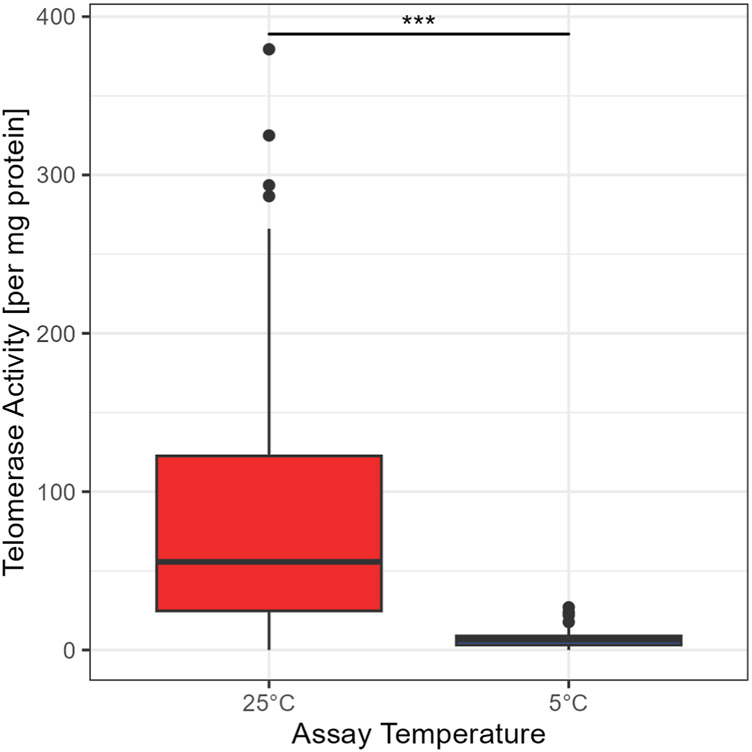
Comparison between TRAP assay concentrations at 25°C and 5°C, for 99 animals in the 25°C group and 105 animals in the 5°C group, the boxes show the 25th percentile, the median, and the 75th percentile (Interquartile Range/IQR), whiskers represent the 25th/75th percentile −/+ 1.5 * IQR; points outside of that range are outliers, asterisks indicate significant differences between groups: ‘***’ <0.001 ‘**’ <0.01 ‘*’ <0.05.

The standard temperature to assess TA is 25°C (Ludlow et al., 2014). To test if telomerase from garden dormice is able to elongate the substrate at low temperatures (comparable to the body temperature during deep torpor), we used the elongation temperature of 5°C (see Figure 1). A Wilcoxon rank test confirmed a significant difference between the two temperatures (W = 9,907, *p* < 0.01). Thus, even if telomerase is present at low temperatures common during deep torpor in garden dormice, TA is minimal and telomere elongation is unlikely to occur via this mechanism. All further comparisons are for TA at 25°C.

**FIGURE 2:**
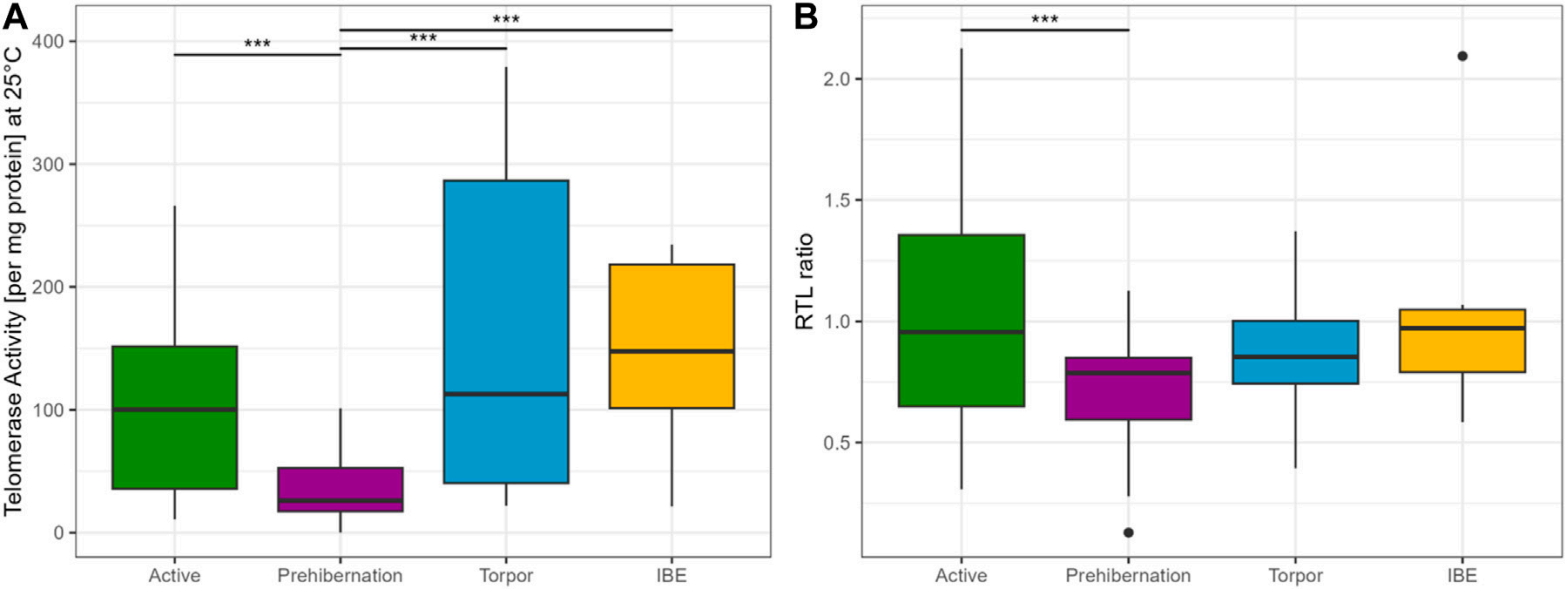
**(A)** TRAP assay concentration at 25°C for all four different seasons, showing that telomerase is present in all phases of the cycle and potentially active. Results represent the glm model (Gaussian distribution), with the response variable telomerase activity at 25°C (square root transformed) and state as the only explanatory variable. For visualisation purposes the plot is on the original scale representing the raw data. **(B)** RTL ratio for all four different seasons. Active and prehibernation seasons only include adult female and male garden dormice, torpor and IBE timepoints only include juvenile males. We cannot compare the different timepoints in a full model, due to these constraints. The R package emmeans (Lenth, 2023) was used for comparisons between groups, asterisks indicate significant differences: “***” <0.001 “**” <0.01 “*” <0.05. The boxes show the 25th percentile, the median, and the 75th percentile (Interquartile Range/IQR), whiskers represent the 25th/75th percentile −/+ 1.5 * IQR, points outside of that range are outliers.

A comparison between the four different seasons showed lower TRAP activity in active and prehibernation phases, compared to Torpor and IBE (see Figure 2A). Be aware though, that in torpor and IBE animals were not older than 10 months, namely, “juveniles”.

**FIGURE 3:**
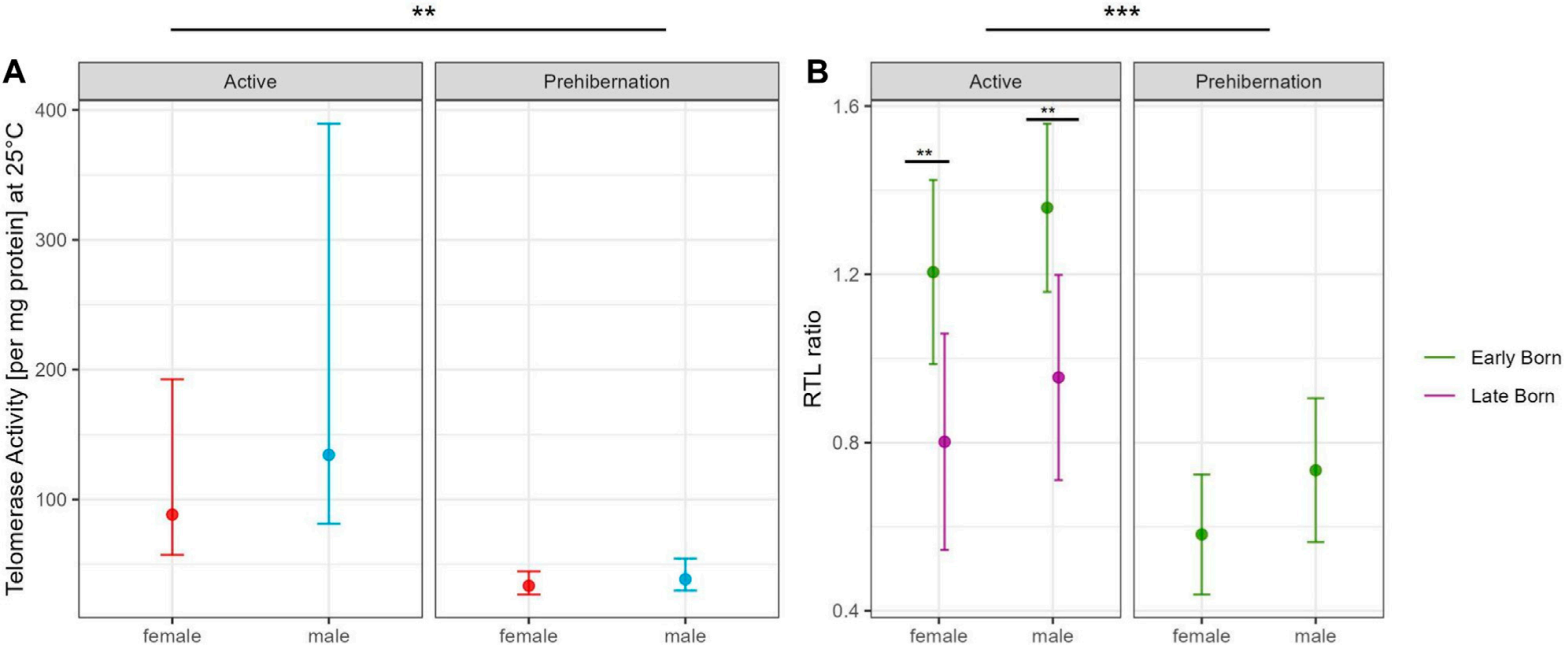
Comparison of **(A)** Partial residual plot of the effect of male (blue) and female (red) adult garden dormice on the TA at 25°C compared between the active and prehibernation season. The underlying model accounts for the effects of body mass, early/late born and age of the animals in months and **(B)** Partial residual plot of the effect of early (green) and late (violet) born garden dormice on RTL comparing the active and prehibernation season, underlying model accounts for the effects of body mass and age of the animals in months. Predictions are back-transformed to the original response scale, whiskers show the standard errors, for each group on square root scale for the telomerase activity plot, asterisks indicate significant differences between groups: “***” <0.001 “**” <0.01 “*” <0.05.

The generalized linear model with TRAP assay at 25°C in the adult dormice group showed that the difference between active and prehibernation season had the strongest effect on telomerase activity per mg protein, with values being higher in the active season compared to prehibernation (Figure 3A, Supplementary Tables S1, 2). All other predictors had no meaningful influence in the model (Supplementary Tables S1, 2).

The model for RTL in adult individuals, with the same predictors as in the TRAP model, showed that seasonal state (active/prehibernation) had the same very strong effect (see Figure 2B) with animals in prehibernation having much shorter RTL than those in the active season (all early born). However, for this model, also the sex of the animals was important with males having longer RTL compared to females in both seasons. The late born individuals (only measured in the active season) had much shorter RTL compared to animals born early in the season (Figure 3B, Supplementary Tables S3, 4). All other variables, body mass and the age of the animals did not have an influence on the model (Supplementary Tables S3, 4).

None of the predictor variables for the models that included the measurements of juvenile males during torpor and IBE (body mass and the feeding regime) had any meaningful influence for the TRAP assay or the RTL ratio. The only variable that stood out, but still had no relative variable importance over 0.7, was body mass in the model including the TRAP assay measurements (Supplementary Tables S5–8).

## Discussion

The clearest and most surprising result from our study is that telomerase is detectable and is maintained at relatively high levels not only during the early active season but also during the two hibernation sampling points, including deep torpor. This runs contrary to our original hypothesis that telomerase activation is costly and unable to be sustained during periods of low energy expenditure such as torpor. It is possible, however, that the TA that we detected is a carry-over of telomerase activated in the period of IBE and, although present, is not active at the low body temperatures experienced during torpor. Our finding that TA is significantly lower when the TRAP phase of the assay is run at 5°C compared to 25°C supports this argument. It is important to note, however, that TA is much lower at 5°C but it was not completely absent (see Figure 1), suggesting that some telomere elongation may still be possible even at such a low temperature. An estimated half-life for the telomerase complex of just 24 h underscores that residual telomerase created during IBE may not be sufficient to maintain activity during deep torpor and that new expression of hTERT would be needed (Holt et al., 1997). This leaves open the intriguing possibility that telomerase can be activated and some telomere elongation is still possible even during torpor, an idea supported by previous research showing an increase in RTL for some individuals over torpor and the hibernation season (Turbill et al., 2013; Giroud et al., 2014). That telomerase is active during IBE is also a novel finding (see Figure 2A) and may shed further light on the need for all hibernating species to periodically return to euthermia. Further research is needed to determine whether TA induced during IBE is purely a consequence of the intense and damaging ROS output generated in this phase of hibernation or a necessary requirement to maintain telomeres as they gradually erode across hibernation. Keeping telomere lengths above a certain threshold and avoiding extreme shortening of telomeric DNA might represent an important strategy in terms of cancer avoidance in animal species living in highly seasonal environments.

Our results show a clear seasonal effect for RTL in garden dormice that has previously been reported in this species (Giroud et al., 2014; Nowack et al., 2019; Giroud et al., 2023) and other species (Hoelzl et al., 2016a; Foley et al., 2018), with a depletion during the hibernation period that is quickly recovered in the active season when food resources are plentiful. This result fits well with our original expectations of a cyclical relationship between RTL and season in the garden dormouse. In the active season, there was also a strong influence of early/late born individuals on RTL. For both sexes, late born individuals had significantly shorter RTL than early born. This result is in line with the costs of catch-up growth previously reported for late born garden dormice (Giroud et al., 2014; Mahlert et al., 2018) and likely reflects a carry-over signature of telomere loss from more frequent IBE phases in their first season of hibernation. We also detected, however, a tendency for RTL to decrease immediately prior to hibernation at a time when individuals are investing intensively in accruing body fat for the winter period (see Figure 2B). This interesting finding confirms that the early active season is the important timepoint for individuals to invest in telomere repair and that, directly before hibernation, energy seems to be diverted to other processes such as lipogenesis and fat storage. It may be, however, that this effect is specific to liver tissue which is the prime organ involved in lipid metabolism and storage. Further tests on other tissue types will help to resolve this question. Interestingly, in our study we were also able to show a corresponding and marked drop in TA at the prehibernation timepoint, which strongly suggests a central role for telomerase in telomere maintenance in this species of hibernating rodent. This finding is incredibly interesting and has important consequences for our understanding of somatic maintenance in general and the energetic costs of telomere repair in particular.

Telomere repair mechanisms have long been postulated to be active in many species (Smith et al., 2022) but the exact nature of these mechanisms has been unclear to date. While telomerase has been the prime candidate, other mechanisms such as ALT (alternative lengthening of telomeres) have also been suggested (Neumann et al., 2013; Shay and Wright, 2019). We show here that, in garden dormice at least, telomerase is closely linked to telomere maintenance and when TA drops we can detect a corresponding drop in RTL. Given the generally high levels of TA at most sampling points however, it is possible that telomerase has other non-canonical functions in this species (Arndt and MacKenzie, 2016). It has been suggested previously that telomerase may be involved in processes as diverse as mitochondrial function (Ahmed et al., 2008; Haendeler et al., 2009; Moslehi et al., 2012), reproduction (Hapangama et al., 2008; Tan et al., 2012; Čapková Frydrychová, 2023), and immunity (Allsopp et al., 2002; Blasco, 2002). Garden dormice may be a good model for investigating such non-canonical functions given the high activity we have detected in this study and their life history which is strongly linked to seasonal fluctuations in energy availability, temperature, and predator avoidance.

Only juvenile male individuals were available for TA measurement during hibernation in this study and they had similar levels of activity to adults in the active season, which was clearly higher than adults in the prehibernation season. This result should be treated with caution given that no juveniles were included in the early active or the prehibernation group. As significant telomere shortening in the first year of life is reported in many species (Monaghan and Ozanne, 2018), it may be that, for juveniles, telomerase is activated at a relatively early timepoint to counteract this loss and TA could be maintained at a high level throughout the active season right up to entering the first hibernation. Our model also highlighted a sex difference in RTL throughout the entire active season (early and immediately prior to hibernation). This finding hints towards a gender biased difference in energy balance during the active season perhaps as a result of reproductive effort. Indeed, male energy requirements for reproduction differ in timing to females in the closely related species *Glis glis*. In males of that species, a large energy demand is related to gonadal development immediately prior and after emergence from hibernation, whereas in females, the main cost is associated with birth and lactation during the main active season (Lebl et al., 2010). The difference in TA that we detected in the active season may reflect an increased investment by females into reproduction and a trade-off in energy balance away from telomere repair. A corresponding trade-off in males likely comes earlier in the active season, immediately prior or right at emergence, and was therefore not captured with our sampling regime. Further studies with additional sampling points should help clarify this issue.

## Data Availability

The raw data supporting the conclusion of this article will be made available by the authors, without undue reservation.
