# Supplementary material for: Seasonal variation in telomerase activity and telomere dynamics in a hibernating rodent, the garden dormouse (Eliomys quercinus)
Source: Front Physiol. 2023 Nov 22;14:1298505. doi: 10.3389/fphys.2023.1298505 (PMC10698472; doi:10.3389/fphys.2023.1298505)
Supplement: Supplementary file 1 [file Table1.pdf]

Supplementary information

Supplementary Table 1: Model selection of the three best models with a delta AIC <2 for the glm model (Gamma(link = "inverse")) with the **TRAP assay** at 25°C **in adult garden dormice.**

Intercept	Age in months	Body mass [g]	Early-late born	Sex	State	df	AICc	delta	weight
0.0097					+	3	733.1	0.00	0.215
0.0118				+	+	4	733.2	0.10	0.205
0.0139		-0,000039			+	4	734.9	1.83	0.086

Supplementary Table 2: “Relative Variable Importance (RVI)" and summary statistics of the best models in model selection for the **TRAP assay** at 25°C **in adult garden dormice** showing the relative influence of included variables.

Variable	Estimate	Standard Error	Relative Variable Importance
Intercept	0.01391	0.006634	/
**State [PREHIBERNATION]**	**0.01848**	**0.004831**	**1.00**
Body mass [g]	-0.00001464	0.00005978	0.26
Sex [Male]	-0.003887	0.002524	0.47
Age in months	-0.00001365	0.00007242	0.24
Early-late [LB]	-0.0002337	0.003067	0.24

Supplementary Table 3: Model selection of the three best models with a delta AIC <2 for the glm model with gaussian distribution with the **RTL ratio in adult garden dormice.**

Intercept	Age in months	Body mass [g]	Early-late born	Sex	State	df	AICc	delta	weight
1.083			+	+	+	5	50.7	0.00	0.314
0.896		0.002	+	+	+	6	51.8	1.07	0.184
1.182	-0.0021		+	+	+	6	52.5	1.82	0.126

Supplementary Table 4: "Relative Variable Importance (RVI)" and summary statistics of the best models in model selection for the **RTL ratio in adult garden dormice** showing the relative influence of included variables.

Variable	Estimate	Standard Error	Relative Variable Importance
Intercept	0.997444	0.218445	/
**State [PREHIBERNATION]**	**-0.568923**	**0.004831**	**1.00**
Body mass [g]	0.002250	0.001828	0.45
**Sex [Male]**	**0.141721**	**0.079612**	**0.76**
Age in months	-0.002675	0.002972	0.30
**Early-late [LB]**	**-0.379595**	**0.133731**	**0.93**

Supplementary Table 5: Model selection of the three best models with a delta AIC <2 for the glm model with a gaussian distribution with the **TRAP assay** at 25°C **in juvenile male garden dormice.**

Intercept	Body mass [g]	State	Feeding regime	df	AICc	delta	weight
536.0	-4.603			3	295.0	0.00	0.341
151.5				2	296.0	0.93	0.214
605.1	-5.126		+	4	296.6	1.53	0.158

Supplementary Table 6: “Relative Variable Importance (RVI)" and summary statistics of the best models in model selection for the **TRAP assay** at 25°C **in juvenile male garden dormice** showing the relative influence of included variables.

Variable	Estimate	Standard Error	Relative Variable Importance
Intercept	606.601	217.528	/
State [TORPOR]	24.974	42.360	0.21
Body mass [g]	-5.303	2.542	0.63
Feeding regime [FASTED]	2.542	42.843	0.29

Supplementary Table 7: Model selection of the two best models with a delta AIC <2 for the glm model with gaussian distribution with the **RTL ratio in juvenile male garden dormice,** be aware that the best model was the null model.

Intercept	Body mass [g]	State	Feeding regime	df	AICc	delta	weight
1.079				2	16.8	0.00	0.400
1.013		+		3	18.6	1.76	0.166

Supplementary Table 8: “Relative Variable Importance (RVI)" and summary statistics of the best models in model selection for the **RTL ratio in juvenile male garden dormice** showing the relative influence of included variables, in this case no variable had an important influence on the model.

Variable	Estimate	Standard Error	Relative Variable Importance
Intercept	0.439217	0.836559	/
State [TORPOR]	0.121583	0.157534	0.279
Body mass [g]	0.006418	0.010022	0.250
Feeding regime [FASTED]	0.099861	0.158393	0.241

Supplementary Table 9: Raw data of all animals included in the study.

ID	Sex	state	Food regime	Early [EB]/ Late Born [LB]	Body Mass [g]	Age Category	Age months	TRAP concentration 5°C	TRAP concentration 25°C	Sampling Time Point
3921	male	Active	*ad libitum*	Early Born	100.4	Adult	26	1.59	-	July-22
3926	female	Active	*ad libitum*	Early Born	77.5	Adult	26	6.28	99.65	July-22
3990	female	Active	*ad libitum*	Early Born	80.4	Adult	26	4.49	222.26	July-22
4064	male	Active	*ad libitum*	Early Born	82.8	Adult	26	6.29	131.80	July-22
4071	female	Active	*ad libitum*	Late Born	78.5	Adult	11	5.81	55.70	July-22
4072	female	Active	*ad libitum*	Late Born	91.2	Adult	11	9.01	10.81	July-22
4073	female	Active	*ad libitum*	Late Born	85.5	Adult	11	7.23	121.04	July-22
4074	male	Active	*ad libitum*	Late Born	103.5	Adult	11	6.71	158.94	July-22
4075	female	Active	*ad libitum*	Late Born	96.1	Adult	11	2.08	28.97	July-22
4078	female	Active	*ad libitum*	Late Born	103.8	Adult	11	2.31	44.38	July-22
4079	female	Active	*ad libitum*	Late Born	84.8	Adult	11	4.64	49.32	July-22
4080	male	Active	*ad libitum*	Late Born	134.4	Adult	11	8.76	102.30	July-22
4118	male	Active	*ad libitum*	Early Born	149.2	Adult	26	11.61	26.24	July-22

4140	female	Active	*ad libitum*	Early Born	68.6	Adult	26	6.41	34.49	July-22
4199	female	Active	*ad libitum*	Early Born	76	Adult	26	7.41	124.11	July-22
4214	male	Active	*ad libitum*	Early Born	105.9	Adult	26	7.9	158.94	July-22
5962	male	Active	*ad libitum*	Late Born	121.8	Adult	11	3.82	149.17	July-22
9078	male	Active	*ad libitum*	Early Born	104.2	Adult	62	15.41	58.63	July-22
9086	male	Active	*ad libitum*	Early Born	96.5	Adult	62	10.07	100.47	July-22
9088	female	Active	*ad libitum*	Early Born	98.1	Adult	62	0	35.18	July-22
9089	female	Active	*ad libitum*	Early Born	99.4	Adult	62	6.28	18.21	July-22
9090	male	Active	*ad libitum*	Early Born	107.6	Adult	62	21.97	177.03	July-22
9410	female	Active	*ad libitum*	Late Born	161.1	Adult	47	17.67	166.80	July-22
9412	male	Active	*ad libitum*	Late Born	103.1	Adult	47	11.88	25.30	July-22
9422	female	Active	*ad libitum*	Early Born	88.4	Adult	62	7.78	36.00	July-22
9423	female	Active	*ad libitum*	Early Born	100.9	Adult	62	14.84	-	July-22

9424	female	Active	*ad libitum*	Early Born	122.6	Adult	62	5.85	129.40	July-22
9428	female	Active	*ad libitum*	Early Born	152.5	Adult	62	6.97	81.48	July-22
9429	female	Active	*ad libitum*	Early Born	103.6	Adult	62	6.32	26.41	July-22
9433	male	Active	*ad libitum*	Early Born	103	Adult	62	17.13	266.06	July-22
9434	female	Active	*ad libitum*	Early Born	109.4	Adult	62	6.94	103.87	July-22
9828	male	Active	*ad libitum*	Late Born	127.9	Adult	47	5.38	257.37	July-22
9910	female	Active	*ad libitum*	Late Born	108.6	Adult	47	14.83	66.40	July-22
9950	male	Active	*ad libitum*	Late Born	114.4	Adult	47	16.03	219.27	July-22
3924	male	IBE	*ad libitum*	Early Born	73.9	Juveniles	10	6.75	234.48	March-20
3935	male	Torpor	*ad libitum*	Early Born	93	Juveniles	10	2.33	112.78	March-20
3937	male	IBE	*ad libitum*	Early Born	83.4	Juveniles	10	-	133.52	March-20
3941	male	Torpor	fasted	Early Born	91.7	Juveniles	10	2.36	21.89	March-20
3943	male	Torpor	*ad libitum*	Early Born	80.5	Juveniles	10	11.83	379.39	March-20

3951	male	Torpor	*ad libitum*	Early Born	82.6	Juveniles	10	4.26	35.02	March-20
3977	male	IBE	fasted	Early Born	95	Juveniles	10	2.27	21.35	March-20
3980	male	Torpor	*ad libitum*	Early Born	108.5	Juveniles	10	2.46	60.55	March-20
3989	male	IBE	*ad libitum*	Early Born	94.8	Juveniles	10	0	222.75	March-20
3992	male	IBE	*ad libitum*	Early Born	78.6	Juveniles	10	0	101.20	March-20
3994	male	Torpor	*ad libitum*	Early Born	89.5	Juveniles	10	2.45	88.69	March-20
3995	male	Torpor	fasted	Early Born	72.8	Juveniles	10	17.32	244.73	March-20
3996	male	IBE	fasted	Early Born	89.1	Juveniles	10	3.42	232.15	March-20
3997	male	IBE	fasted	Early Born	70.6	Juveniles	10	3.44	104.21	March-20
3999	male	IBE	*ad libitum*	Early Born	82.7	Juveniles	10	2.47	205.00	March-20
4000	male	Torpor	*ad libitum*	Early Born	78.1	Juveniles	10	6.86	286.63	March-20
4081	male	Torpor	*ad libitum*	Early Born	73.3	Juveniles	10	4.99	-	March-20
4082	male	IBE	*ad libitum*	Early Born	79.8	Juveniles	10	5.19	-	March-20

4083	male	Torpor	fasted	Early Born	79.4	Juveniles	10	17.08	325.00	March-20
4085	male	Torpor	fasted	Early Born	79.2	Juveniles	10	13.82	40.42	March-20
4087	male	Torpor	fasted	Early Born	84	Juveniles	10	3.04	24.92	March-20
4088	male	IBE	fasted	Early Born	76.5	Juveniles	10	5.42	101.62	March-20
4115	male	Torpor	*ad libitum*	Early Born	81.8	Juveniles	10	6.43	144.05	March-20
4118	male	IBE	fasted	Early Born	84.9	Juveniles	10	0.3	31.39	March-20
4119	male	Torpor	fasted	Early Born	76.8	Juveniles	10	4.12	293.46	March-20
4200	male	IBE	*ad libitum*	Early Born	77.6	Juveniles	10	3.17	191.05	March-20
4212	male	Torpor	*ad libitum*	Early Born	82.6	Juveniles	10	-	143.09	March-20
3965	female	Prehibernation	*ad libitum*	Early Born	162.2	Adult	41	3.67	7.23	October-22
3966	male	Prehibernation	*ad libitum*	Early Born	183.4	Adult	41	0.00	17.04	October-22
3969	female	Prehibernation	*ad libitum*	Early Born	157	Adult	41	2.94	9.38	October-22
4011	male	Prehibernation	*ad libitum*	Early Born	142.2	Adult	41	1.10	10.72	October-22

4014	female	Prehibernation	*ad libitum*	Early Born	177.9	Adult	41	0.95	53.78	October-22
4017	male	Prehibernation	*ad libitum*	Early Born	177.9	Adult	41	2.11	65.71	October-22
4019	male	Prehibernation	*ad libitum*	Early Born	151.3	Adult	41	4.28	18.16	October-22
4020	male	Prehibernation	*ad libitum*	Early Born	169.3	Adult	41	11.95	18.89	October-22
4065	female	Prehibernation	*ad libitum*	Early Born	126.2	Adult	41	4.39	-	October-22
4067	male	Prehibernation	*ad libitum*	Early Born	203.9	Adult	41	8.48	17.47	October-22
4069	male	Prehibernation	*ad libitum*	Early Born	131.9	Adult	41	27.01	104.77	October-22
4188	male	Prehibernation	*ad libitum*	Early Born	176.1	Adult	41	1.93	-	October-22
9408	male	Prehibernation	*ad libitum*	Early Born	145	Adult	53	6.61	17.94	October-22
9413	male	Prehibernation	*ad libitum*	Early Born	185.4	Adult	53	5.46	4.32	October-22
9414	male	Prehibernation	*ad libitum*	Early Born	144.2	Adult	53	5.76	19.94	October-22
9837	male	Prehibernation	*ad libitum*	Early Born	158.9	Adult	53	23.79	22.56	October-22
9835	male	Prehibernation	*ad libitum*	Early Born	190.3	Adult	53	2.30	16.43	October-22

9902	male	Prehibernation	*ad libitum*	Early Born	171.6	Adult	53	2.57	27.92	October-22
9912	female	Prehibernation	*ad libitum*	Early Born	122	Adult	53	2.27	52.12	October-22
9947	male	Prehibernation	*ad libitum*	Early Born	153.8	Adult	53	3.68	100.96	October-22
9954	female	Prehibernation	*ad libitum*	Early Born	137.9	Adult	53	0.79	1.48	October-22
9956	female	Prehibernation	*ad libitum*	Early Born	155.5	Adult	53	0.00	6.84	October-22
9962	female	Prehibernation	*ad libitum*	Early Born	124.7	Adult	53	6.90	17.62	October-22
9963	male	Prehibernation	*ad libitum*	Early Born	130.1	Adult	53	14.17	23.6	October-22
3961	female	Prehibernation	*ad libitum*	Early Born	147.8	Adult	41	5.50	101.29	October-22
3962	male	Prehibernation	*ad libitum*	Early Born	177.9	Adult	41	5.49	68.75	October-22
3963	female	Prehibernation	*ad libitum*	Early Born	161	Adult	41	3.95	36.44	October-22
3967	female	Prehibernation	*ad libitum*	Early Born	152.1	Adult	41	8.65	82.77	October-22
3968	female	Prehibernation	*ad libitum*	Early Born	156.9	Adult	41	9.14	82.81	October-22
4030	female	Prehibernation	*ad libitum*	Early Born	154	Adult	41	8.24	60.64	October-22

4061	male	Prehibernation	*ad libitum*	Early Born	173.2	Adult	41	5.44	89.83	October-22
4063	female	Prehibernation	*ad libitum*	Early Born	120.1	Adult	41	11.10	-	October-22
4068	male	Prehibernation	*ad libitum*	Early Born	188.4	Adult	41	10.27	37.11	October-22
4012	female	Prehibernation	*ad libitum*	Early Born	134.2	Adult	41	8.61	-	October-22
9407	male	Prehibernation	*ad libitum*	Early Born	135.1	Adult	53	2.94	31.66	October-22
9409	female	Prehibernation	*ad libitum*	Early Born	121.9	Adult	53	8.78	40.41	October-22
9420	female	Prehibernation	*ad libitum*	Early Born	113.3	Adult	53	13.78	23.59	October-22
9832	female	Prehibernation	*ad libitum*	Early Born	161.4	Adult	53	12.97	52.26	October-22
9836	male	Prehibernation	*ad libitum*	Early Born	170.2	Adult	53	7.08	20.36	October-22
9908	female	Prehibernation	*ad libitum*	Early Born	153.2	Adult	53	12.40	0	October-22
9915	female	Prehibernation	*ad libitum*	Early Born	137.6	Adult	53	8.47	24.4	October-22
9946	female	Prehibernation	*ad libitum*	Early Born	126.7	Adult	53	7.51	72.75	October-22
9951	male	Prehibernation	*ad libitum*	Early Born	121.9	Adult	53	8.85	30.15	October-22

9953	female	Prehibernation	*ad libitum*	Early Born	116.7	Adult	53	10.40	41.03	October-22
9955	male	Prehibernation	*ad libitum*	Early Born	178.8	Adult	53	8.21	14.25	October-22
9959	female	Prehibernation	*ad libitum*	Early Born	157.7	Adult	53	7.44	44.33	October-22
